# Dynamic Association of Mortality Hazard with Body Shape

**DOI:** 10.1371/journal.pone.0088793

**Published:** 2014-02-20

**Authors:** Nir Y. Krakauer, Jesse C. Krakauer

**Affiliations:** 1 Department of Civil Engineering, The City College of New York, New York, New York, United States of America; 2 Alzohaili Medical Consultants, Southfield, Michigan, United States of America; Tulane School of Public Health and Tropical Medicine, United States of America

## Abstract

**Background:**

A Body Shape Index (ABSI) had been derived from a study of the United States National Health and Nutrition Examination Survey (NHANES) 1999–2004 mortality data to quantify the risk associated with abdominal obesity (as indicated by a wide waist relative to height and body mass index). A national survey with longer follow-up, the British Health and Lifestyle Survey (HALS), provides another opportunity to assess the predictive power for mortality of ABSI. HALS also includes repeat observations, allowing estimation of the implications of changes in ABSI.

**Methods and Findings:**

We evaluate ABSI z score relative to population normals as a predictor of all-cause mortality over 24 years of follow-up to HALS. We found that ABSI is a strong indicator of mortality hazard in this population, with death rates increasing by a factor of 1.13 (95% confidence interval, 1.09–1.16) per standard deviation increase in ABSI and a hazard ratio of 1.61 (1.40–1.86) for those with ABSI in the top 20% of the population compared to those with ABSI in the bottom 20%. Using the NHANES normals to compute ABSI z scores gave similar results to using z scores derived specifically from the HALS sample. ABSI outperformed as a predictor of mortality hazard other measures of abdominal obesity such as waist circumference, waist to height ratio, and waist to hip ratio. Moreover, it was a consistent predictor of mortality hazard over at least 20 years of follow-up. Change in ABSI between two HALS examinations 7 years apart also predicted mortality hazard: individuals with a given initial ABSI who had rising ABSI were at greater risk than those with falling ABSI.

**Conclusions:**

ABSI is a readily computed dynamic indicator of health whose correlation with lifestyle and with other risk factors and health outcomes warrants further investigation.

## Introduction

A Body Shape Index (ABSI) was developed as a way to quantify the risk associated with abdominal obesity, as indicated by a wide waist relative to height and body mass index (BMI) [1]. Above average ABSI was associated with substantially higher risk of death in the United States (USA) National Health and Nutrition Examination Survey (NHANES) 1999–2004 sample, and the excess mortality hazard associated with high ABSI was greater than that associated with above-average body mass index (BMI) or waist circumference (WC) [1]. Several studies have sought to quantify associations between ABSI and adverse outcomes other than death, including diabetes [2], metabolic syndrome [3], and high blood pressure [4], [5]. ABSI seems a promising metric of health risk associated with abdominal adiposity [6].

However, several limitations of the NHANES sample were identified [1]. (1) Hip circumference was not measured, so ABSI could not be directly compared to waist-to-hip ratio (WHR), a popular alternative measure of body shape. Waist to height ratio (WHtR) was also not directly evaluated against ABSI as a predictor of mortality. (2) Anthropometric measurements were only taken on one occasion for each individual, so it is unknown to what extent ABSI varies over time and whether, for given initial ABSI, change in ABSI affects mortality risk. (3) Follow-up was relatively short (averaging 5 years), so that it was not shown that ABSI continues to be a predictor of risk with longer follow-up.

The Health and Lifestyle Survey (HALS) provides an opportunity to examine these questions in a different large national sample, in this case from Britain, thus extending our understanding of ABSI as a predictor of mortality hazard. We build on recent work that found ABSI to be a robust predictor of mortality hazard in HALS [7].

This paper has the following structure: (1) We briefly introduce the HALS data and the analysis methods we adopt; (2) we analyze mortality hazard as a function of ABSI in HALS, with comparison to widely used anthropometric measures, including WHR; (3) we evaluate mortality hazard as a function of change in ABSI between the 2 HALS examinations spaced 7 years apart and the evolution of ABSI-associated risk over the follow-up period.

## Methods

### HALS Data

The first Health and Lifestyle Survey (HALS1) was carried out between September 1984 and June 1985. HALS1 was designed to sample adults (age 18 and over) living in private households in Great Britain (England, Wales, and Scotland) [8]. Addresses were randomly selected from electoral registers using a three-stage design. One adult resident was randomly selected from each chosen address. HALS1 included a comprehensive interview covering health-related attitudes and behaviors; clinical measurements made by a nurse; and a self-completion questionnaire for psychological testing. In total, 7414 individuals completed the interview and the clinical measurements. The sample was broadly representative of the British population in terms of region, employment status, national origin, and age. Compared to the national census there was a slight excess of women in their 20 s to 40 s and men older than 60, presumably because these age groups were more likely to be found at home [8]. Clinical measurements included height, weight, and girth (WC); these were not measured for pregnant women or for those missing limbs. WC was measured with a plastic tape midway between the lowest rib and the top of the hip [9]. Hip circumference at the top of the iliac crest was also measured for most of the sample.

The Health and Lifestyle Survey: Seven-Year Follow-Up (HALS2) was carried out in September 1991 to October 1992. HALS2 re-surveyed and measured, using similar protocols, all HALS1 participants who could be traced [10]. Further, National Health Service records have been used to identify deaths and cancer cases in HALS participants through June 2009, representing over 24 years of follow-up from the beginning of HALS1.

We obtained public-use versions of the HALS data, including anthropometric measurements and follow-up for mortality, from the United Kingdom Data Service, which has processed the data for quality control and to remove personally identifying information [11]–[13]. All protocols for HALS were approved by the British Medical Association Ethical Committee. Participant informed consent for the clinical measurements was recorded in writing [8], [10].

A total of 7011 individuals in our sample had height, weight, and WC data from HALS1 together with follow-up for mortality available (2203 deaths recorded).

### Analysis Approaches

We used Cox proportional hazard regression with age as the timescale [1], [14] to correlate all-cause mortality from the HALS sample with ABSI and with other variables, including BMI, WC, WHR, and WHtR. For each regression, sex was also included as an explanatory variable. Analyses were implemented in the computer language R, using the survival package for proportional hazard regression [15].

ABSI is defined as (Equation 3 in [1]):
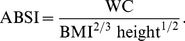
(1)


If SI units are used (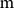 for height and WC, 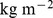 for BMI), the units of ABSI are 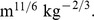 The ABSI z score is defined as (Equation 4 in [1]):
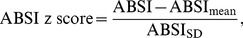
(2)where 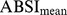 and 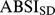 designate age- and sex-specific population means and standard deviations. We have tabulated 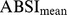 and 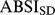 estimated from the NHANES 1999–2004 population [1]. For the present analysis of the HALS population, we generated normals (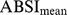 and 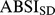) for both the NHANES 1999–2004 and for HALS1 using smoothing spline fits to the mean and standard deviation of ABSI by age and sex, similar to [1]. We considered as predictors ABSI z scores computed using the HALS normals (denoted ABSI (H)) as well as those computed using the NHANES normals (denoted ABSI (N)). The latter affords more direct comparison to the previous analysis of NHANES [1]. All other anthropometric variables were converted to z scores using normals obtained from the HALS1 population with the same procedure as for ABSI (H).

The hazard ratios obtained from our Cox proportional hazard regressions quantify the mortality risk elevation per standard deviation increase in each predictor variable, but cannot be readily compared to determine which variable is the best predictor of mortality. To quantitatively evaluate whether ABSI is a better predictor of mortality hazard than related anthropometric metrics such as WHtR and WHR, we compared the Akaike information criterion (AIC) scores from each regression [16]. The AIC are scaled to produce AIC difference scores Δ*_i_* For the best performing model (with lowest AIC) 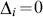, while all other models have positive Δ*_i_* [16]. The likelihood of each model given the data is proportional to 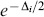 [16]. Thus, we considered 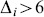 to indicate models that perform significantly worse than the best-performing model (at the 95% confidence level) as mortality predictors for the sampled population (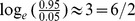). We also calculated coefficients of determination *R* ^2^, denoting the proportion of variation in mortality explained by the predictors of each model, relative to a reference model containing only age and sex as predictors [17].

The NHANES 1999–2004 sample had available mortality follow-up for only a median of 5 years [1]. To evaluate the ability of ABSI to predict mortality hazard at longer lead times, we also used Cox proportional hazard regression on 5-year slices of the 24-year HALS follow-up. For each 5-year period, the regression was performed on those who were alive at the beginning of the period, and the outcome considered was limited to deaths that occurred during the period.

To place this study in the context of our previous work with NHANES, we compared population normals for ABSI and other body measures (height, weight, WC, BMI) between the two studies, and also compared mortality hazards as functions of ABSI for the two samples. These comparisons were intended to be informal, as we did not attempt here a meta-analysis that adjusts for differences between the two samples in such attributes as ethnicity and length of follow up.

## Results

### Anthropometric Risk Factors for Mortality in HALS1

**Figure 1:**
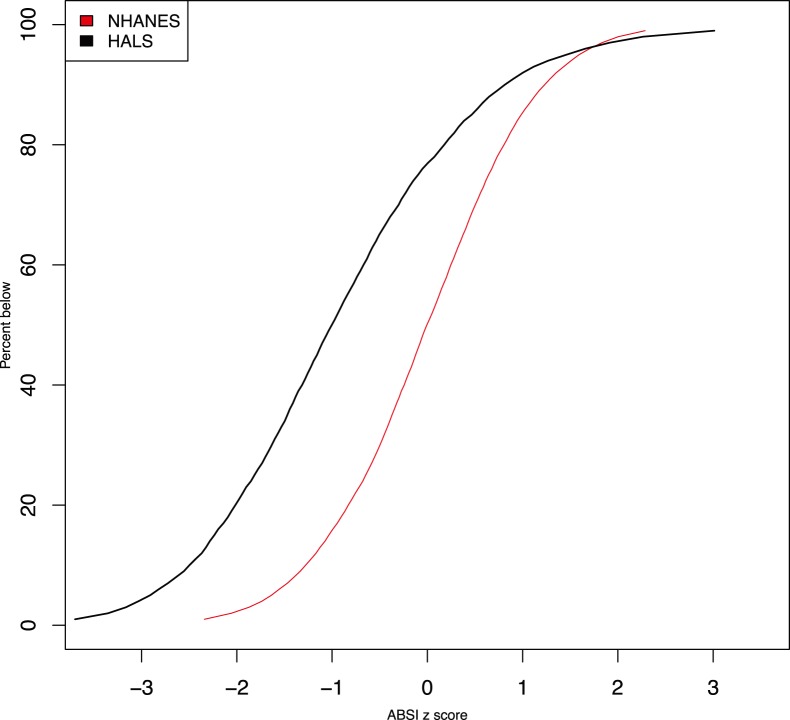
Cumulative distribution function for ABSI z score (calculated using NHANES normals) in the NHANES 1999–2004 compared to the HALS1 samples, showing the mostly lower values of ABSI seen in HALS. For each sample, the 1st through the 99th percentiles are plotted.

The British HALS1 sample was, on average, considerably thinner and narrower-waisted than the later USA NHANES 1999–2004 sample (Table 1). Their mean ABSI was lower than seen in NHANES, meaning that waist circumferences in HALS averaged lower than would have been expected for their weight based on the NHANES sample. The mean ABSI (N) z score, which adjusts for differences in the age and sex composition of the two samples, was lower in HALS by 0.9 of the NHANES population standard deviation (Table 1). The cumulative distribution functions of ABSI z scores (Figure 1) highlights the large fraction of the HALS sample that had smaller ABSI (N) than typically seen in NHANES (z scores 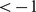), as indicated by the HALS curve being above NHANES for any given ABSI z score. The very highest values of ABSI (z scores 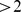) are the exception due to a small fraction of HALS having very high scores (Figure 1). As in NHANES, there was little correlation between ABSI and BMI z scores (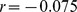 using ABSI(H), or 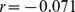 using ABSI(N)).

**Table 1:** HALS and NHANES means of body measures.

	HALS	NHANES
Height (cm)	166.4	167.7
Weight (kg)	68.1	78.7
BMI (kg m^−2^)	24.5	27.9
WC (cm)	82.9	95.8
WHtR	0.499	0.572
ABSI (m^11/6^ kg^−2/3^)	0.0763	0.0808
ABSI z score	−0.90	−0.01

Comparison of mean values in HALS1 and in NHANES 1999–2004 nonpregnant adults. ABSI z scores are based on the NHANES 1999–2004 population normals.

As found for NHANES 1999–2004 [1], ABSI z score was a strong linear predictor of HALS log mortality hazard, indicating that those with higher ABSI faced greater risk of premature death. ABSI (N) and ABSI (H) were comparably good linear predictors, as shown by the similarity of the Akaike information criterion scores (Δ*_i_* in Table 2). WC and WHtR z scores were also significant linear predictors of log mortality hazard in HALS, with WHtR outperforming WC, but, as shown by the respective model Δ*_i_*, both were significantly weaker predictors than ABSI (H) or (N) (Table 2). BMI z score was not a significant linear predictor of mortality hazard (Table 2). The *R* ^2^ values for each model support the ranking of predictor variables given by Δ*_i_*, with ABSI explaining more of the population mortality variability than the other anthropometric measures (Table 2). Given the similarly good performance of ABSI (H) and ABSI (N), all analyses presented below will use the ABSI z scores calculated using NHANES normals (ABSI (N)), unless otherwise specified.

**Table 2:** Mortality hazard association with HALS1 body size and shape.

	Hazard ratio per SD increase	Δ*_i_*	*R* ^2^
ABSI (N)	1.13 (1.09–1.16)	0	0.008
ABSI (H)	1.18 (1.13–1.23)	0.2	0.008
BMI	1.00 (0.96–1.05)	55.6	0.000
WC	1.09 (1.03–1.14)	43.3	0.002
WHtR	1.11 (1.06–1.16)	34.6	0.003

Results of Cox proportional hazard modeling for mortality risk with ABSI, BMI, WC, or WHtR z scores at HALS1 taken as linear predictors. Ranges in parentheses are 95% confidence intervals.

SD = standard deviation; Δ*_i_*  = Akaike information criterion score difference relative to the best performing model shown (see Methods for details); *R* ^2^ = coefficient of determination; ABSI(N) and ABSI(H) refer to ABSI z scores calculated using NHANES or HALS normals, respectively.

Estimating mortality hazard by quintile for ABSI and the other anthropometric measures offers more insight into the relative risk associated with different metrics (Table 3). Relative to the middle quintile, the lower quintiles of ABSI z score had significantly reduced mortality hazard, while the top quintile showed significantly elevated mortality hazard, with 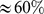 difference in mortality hazard seen between the two extreme quintiles. This hazard elevation for increasing ABSI was similar in magnitude to that seen in NHANES 1999–2004 [1]; the relative mortality hazard for the top quintile compared to the bottom quintile of ABSI z score was 1.61 (95% confidence interval, 1.40–1.86) for HALS compared to 2.04 (1.43–2.92) for NHANES.

**Table 3:** Mortality by quintile.

	Hazard ratio				
Quintile	ABSI (N)	ABSI (H)	BMI	WC	WHtR
1 (lowest)	0.77 (0.66–0.88)	0.83 (0.72–0.95)	1.35 (1.17–1.55)	1.03 (0.90–1.18)	1.01 (0.89–1.16)
2	0.78 (0.68–0.89)	0.92 (0.80–1.05)	1.14 (0.99–1.31)	0.97 (0.85–1.11)	1.00 (0.86–1.14)
3 (reference)	1	1	1	1	1
4	0.97 (0.85–1.11)	1.07 (0.94–1.22)	1.06 (0.93–1.21)	1.03 (0.90–1.17)	1.14 (1.00–1.30)
5 (highest)	1.23 (1.09–1.40)	1.35 (1.18–1.53)	1.23 (1.08–1.39)	1.22 (1.07–1.39)	1.34 (1.18–1.53)

Cox proportional hazard modeling for mortality risk with ABSI, BMI, WC, or WHtR z score quintiles at HALS1 taken as the predictors. Hazard ratios are relative to the middle quintile in each case. Ranges in parentheses are 95% confidence intervals.

ABSI(N) and ABSI(H) refer to ABSI z scores calculated using NHANES or HALS normals, respectively.

The between-quintile cut points are 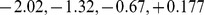 for ABSI (N); 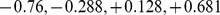 for ABSI (H); 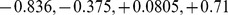 for BMI; 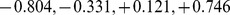 for WC; and 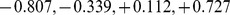 for WHtR.

**Figure 2:**
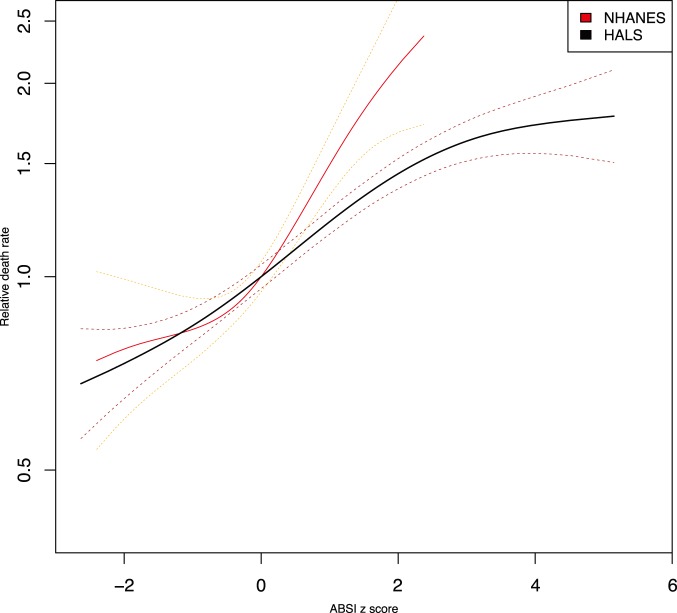
Mortality hazard by ABSI for NHANES 1999–2004 compared to HALS1. Estimates are from proportional hazard modeling where log mortality hazard is a smoothing-spline function in ABSI z score (calculated using NHANES normals). Curves are positioned so that an ABSI z score of 0 has a relative death rate of 1. Dashed curves show 95% confidence intervals. The ranges shown cover the 1st through 99th percentiles of ABSI in each sample. The vertical axis is logarithmic.

Plotting nonparametric smoothing spline regressions of mortality hazard against ABSI z score for both cohorts (Figure 2) again shows that the relative risks found for HALS are similar in magnitude to those seen in NHANES 1999–2004, although the mortality hazard in HALS appears to increase less steeply at high ABSI (z scores 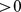) than was seen in NHANES.

WC and WHtR also showed elevated risk at the highest quintile in HALS, though unlike ABSI they did not show significantly reduced risk in the below-average quintiles (Table 3). By contrast, BMI showed the same U-shaped association with mortality hazard seen in NHANES 1999–2004 and in other studies [18], with both the lowest and highest quintiles of the HALS sample subject to elevated risk compared to the middle quintile (Table 3).

Next, we compared ABSI to WHR as a predictor of mortality. (We did not include WHR in comparisons just presented because hip circumference was measured only for part of HALS1, reducing the sample size from 7011 to 4454.) As Table 4 shows, elevated ABSI and WHR were both associated with increased mortality hazard, although the association with ABSI was significantly stronger, as indicated by the model Δ*_i_*. The two indicators were positively correlated (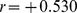 for the z scores), inasmuch as high ABSI and high WHR both indicate a thick-waisted body shape. However, when ABSI and WHR were combined in the same predictive model, ABSI retained its predictive ability while WHR showed no significant association, suggesting that knowing WHR offered no added predictive value given ABSI (Table 4).

**Table 4:** HALS1 mortality hazard association with ABSI versus waist to hip ratio.

	Hazard ratio per SD increase	Δ*_i_*	*R* ^2^
ABSI	1.14 (1.09–1.19)	0	0.007
WHR	1.10 (1.04–1.16)	23.3	0.002
Combined:		2.0	0.007
ABSI	1.14 (1.08–1.20)		
WHR	1.00 (0.93–1.06)		

Results of Cox proportional hazard modeling for mortality risk with ABSI or WHR z scores, or both, taken as linear predictors. Ranges in parentheses are 95% confidence intervals. Only the subsample with hip measurements in HALS1 is used (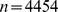; 1408 deaths).

SD = standard deviation; Δ*_i_*  = Akaike information criterion score difference relative to the best performing model shown (see Methods); *R* ^2^ = coefficient of determination.

### Change in ABSI and Predictive Power Over Time

Since anthropometric measurements for much of the sample are available for both HALS1 and HALS2, we could consider how ABSI changed over the intervening 7 years and how this impacted the subsequent mortality risk. Between HALS1 and HALS2, BMI and waist circumference on average increased by about 0.3 standard deviations even after controlling for aging by using z scores (Table 5), a trend also seen over this time period in other countries [19]. ABSI also increased, by about 0.2 standard deviations, suggesting that waistlines were expanding, on average, faster than the 2/3 power of weight used in the definition of ABSI. By contrast, there was little change in mean waist to hip ratio (Table 5). While ABSI increased on average, there was wide variation between individuals, with the standard deviation of the change between HALS1 and HALS2 approaching 1 (Table 5).

**Table 5:** Anthropometry changes between HALS1 and HALS2.

	Mean	SD
ABSI	+0.16	0.98
BMI	+0.27	0.60
WC	+0.26	0.73
WHtR	+0.22	0.72
WHR	−0.02	1.11

Mean and standard deviation (SD) of changes in z scores between HALS1 and HALS2. All z scores are based on HALS1 population normals.

Along with the ABSI z scores at baseline (HALS1) and on 7-year follow up (HALS2), the change in z score between HALS1 and HALS2 (ΔABSI) was a predictor of mortality hazard over the period following HALS2. Increasing ABSI (positive ΔABSI) correlated with greater risk compared to decreasing ABSI (negative ΔABSI) (Table 6). Table 6 also presents several regression models that serve to illustrate the relative mortality associations. Model A shows that the baseline (HALS1) ABSI value and the change over time (ΔABSI) each make similarly significant contributions to mortality hazard. Model B demonstrates that given the later (HALS2) measurements, past change in ABSI was no longer a significant predictor of risk. Finally, Model C shows that, as would be expected, the more recent (HALS2) ABSI data are better at predicting subsequent mortality than the earlier HALS1 measurement.

**Table 6:** Mortality hazard association with ABSI from HALS1 and HALS2.

	Hazard ratio per SD increase
ABSI1	1.09 (1.04–1.14)
ABSI2	1.17 (1.12–1.23)
ΔABSI	1.05 (1.01–1.10)
Combined A:	
ABSI1	1.19 (1.13–1.26)
ΔABSI	1.16 (1.10–1.22)
Combined B:	
ΔABSI	0.97 (0.92–1.02)
ABSI2	1.19 (1.13–1.26)
Combined C:	
ABSI1	1.03 (0.98–1.08)
ABSI2	1.16 (1.10–1.22)

Results of Cox proportional hazard modeling for mortality risk with ABSI1 or ABSI2 (ABSI from HALS1 or HALS2 measurements, respectively) or ΔABSI (ABSI2 minus ABSI1), or combinations thereof, taken as linear predictors. Ranges in parentheses are 95% confidence intervals. Only the subsample with measurements in both HALS1 and HALS2 is used (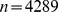; 1094 deaths).

SD = standard deviation.

In contrast to the findings with ABSI, changes in the z scores of the other anthropometric measures considered (BMI, WC, WHtR, WHR) were not significant linear predictors of mortality hazard (results not shown).

Table 7 shows the mortality hazard ratio for the baseline HALS ABSI z score computed for 5 year intervals after study initiation. The mortality risk is significant and of a similar magnitude for each lead-time interval, up to at least 20 years after the baseline measurements (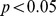 for each 5-year interval, except for 21–24 years, where 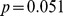).

**Table 7:** Mortality hazard association with HALS1 ABSI z score.

Lead time (y)	*n* at beginning of period	Deaths during period	Hazard ratio per SD increase
0–5	7011	397	1.16 (1.08–1.24)
6–10	6614	470	1.15 (1.07–1.22)
11–15	6144	457	1.09 (1.02–1.17)
16–20	5687	497	1.12 (1.04–1.20)
21–24	5190	382	1.08 (1.00–1.17)

Results of Cox proportional hazard modeling for mortality risk at different lead times with ABSI (N) at HALS1 taken as a linear predictor. Ranges in parentheses are 95% confidence intervals.

SD = standard deviation.

## Discussion and Conclusions

The relatively large, nationally representative HALS sample, resurvey, and long-term follow-up has previously been used to study health behaviors and their association with mortality and cancer hazards. For example, WHtR and WHR were both significant predictors of mortality hazard in the HALS sample, whereas BMI was not [20]. The superiority of WHtR as a mortality predictor in the HALS sample was confirmed by a later study using mortality follow-up to 2006 [21]. An index of poor health behaviors including smoking, inactivity, eating few fruits and vegetables, and overindulgence in alcohol was a strong predictor of mortality hazard in HALS [22]. Here, we extend these findings by showing that the recently developed ABSI is a robust predictor of mortality hazard in HALS, compared to the previously evaluated anthropometric indices.

We found that while waist to hip ratio was associated with mortality, WHR did not improve predictions when included as a covariate with ABSI. Since determining ABSI only requires a waist circumference measurement in addition to the standard height and weight measurements used to determine BMI while WHR additionally requires a hip circumference measurement, ABSI may also be the more clinically convenient of the two metrics. (By this criterion, WHR could be preferred in situations where height and weight measurements are not routinely obtained.) Waist circumference alone, or waist to height ratio, are metrics that require no additional measurements compared to ABSI, but their association with mortality was weaker than that of ABSI. To the extent that these conclusions are borne out by similar analyses carried out on more large samples, this suggests that ABSI is a superior basic anthropometric indicator at least for the risk of premature death. Despite the dynamic element in ABSI, we found that it was sufficiently persistent to predict mortality hazard for at least 20 years after measurement (Table 7).

We used here ABSI z scores, instead of the raw values, as predictors of risk. The reason for this is that ABSI tends to increase strongly with age and to be substantially higher for men than for women [1]. Using unadjusted ABSI as a predictor, as in several recent studies [2]–[4], [23], risks confounding body shape variation with age and sex as risk factors in diverse population samples. Calculation of z scores does add to the complexity of using ABSI in the clinical setting, and whether simpler methods might provide comparable performance is an important topic for future study. Meanwhile, an online calculator of ABSI and its z score (using NHANES normals) is available from us at http://www-ce.ccny.cuny.edu/nir/sw/absi-calculator.html.

One uncertainty in our analysis is that the protocol for measuring WC in HALS was not identical to that in NHANES, which would bias the ABSI z scores for HALS computed using the NHANES normals and which may conceivably have contributed to the difference in ABSI distributions between the two populations (Figure 1). Further quantification of the magnitude of WC measurement differences between methods would clearly be helpful. Nevertheless, the relatively good performance of the NHANES population normals for constructing ABSI z scores for HALS that are near-linear predictors of log mortality hazard (Table 2 and Figure 2) provides some reason to believe that a range of WC measurement protocols may yield broadly comparable values of ABSI. This is consistent with a meta-analysis that found that WC measurement protocol did not influence the associations observed between WC, mortality, cardiovascular disease, and diabetes [24].

For given initial ABSI, we found that change in ABSI over time was a predictor of all-cause mortality hazard, with increasing ABSI associated with increased risk. By contrast, studies have typically not found simple associations between change in BMI and mortality. Intentional weight loss may have better outcomes than unintended weight loss [25]–[29], while a large observational study found that decreasing cardiorespiratory fitness, but not BMI change, increases mortality hazard [30]. Complicating matters further, the recent large randomized controlled trial of type 2 diabetics Look AHEAD found no impact on cardiovascular event or mortality risk from a one-year intensive lifestyle intervention which led to weight loss [31]. We further note that although the current study only examines all-cause mortality as an outcome, the HALS follow-up includes data on cause of death as well as on cancer diagnosis, which could be used to study the health impacts of body shape in more detail.

There is evidence from previous intervention studies that dieting and exercise regimens can under some circumstances decrease waist circumference by proportionally more than weight and thus reduce ABSI [32]–[35]. Thus, it is worth exploring whether the mortality reductions seen in some studies [36], [37] among those intending to lose weight, regardless of actual weight change, could be correlated with reduction in ABSI related to lifestyle change. Change in ABSI could potentially offer a novel, clinically informative indicator of the effectiveness of lifestyle modification. More analysis of population surveys such as HALS could lead to better understanding of lifestyle and other correlates of ABSI change. Data from randomized trials of weight loss such as Look AHEAD could also be used to test whether change in ABSI is predictive of impacts on other risk factors such as metabolic syndrome components and on morbidity and mortality outcomes.

In summary, ABSI, previously found to be a strong risk factor for mortality in a USA population sample (NHANES 1999–2004), has similar associations with mortality hazard in a British sample (HALS). Further, ABSI appears to outperform other popular anthropometry-based measures of adiposity, such as WHtR and WHR. Mortality risk appears to track changes in ABSI over time, motivating further research into whether lifestyle or other interventions could trigger reduction in ABSI and incur the longevity benefits seen in this study for those with lower ABSI.
